# Systematic Review of Self-Assessment Scales for Negative Symptoms in Schizophrenia

**DOI:** 10.3390/brainsci15020148

**Published:** 2025-01-31

**Authors:** Lucie Métivier, Sonia Dollfus

**Affiliations:** 1 UMR-S 1237, Neuropresage Team, GIP Cyceron, 14000 Caen, France; metivier@cyceron.fr; 2 Department of Health (UFR Santé), University of Caen Normandy (UNICAEN), 14000 Caen, France; 3 Academic and Medical Research Federation (FHU A2M2P), University of Caen Normandy (UNICAEN), 14000 Caen, France; 4 Department of Psychiatry, Esquirol Center, University Hospital Center (CHU), 14000 Caen, France

**Keywords:** self-assessment, negative symptoms, schizophrenia

## Abstract

**Background/Objectives**: Negative symptoms (NSs) significantly impair the outcome of schizophrenia, primarily due to their effect on quality of life and their resistance to pharmacological treatments. Several scales have been developed to assess the various dimensions of NSs, including avolition, anhedonia, alogia, social withdrawal, and blunted affect. While observer-rated scales are the most commonly used, self-assessment tools remain underutilized. However, self-assessments offer a promising approach for gaining insights into the personal experiences of individuals. The objective of this review was to identify and report the psychometric properties of self-assessment scales for NSs that are relevant for both research and clinical practice, with a focus on tools that assess multiple domains of NSs in order to support comprehensive evaluations and tailored therapeutic strategies. **Methods**: We conducted an exhaustive literature review following PRISMA guidelines to identify self-evaluation scales that evaluate several domains of NSs in the MEDLINE and Web of Science databases. The COSMIN checklist was used to assess the methodological quality of each tool. **Results**: Our review identified five self-assessment scales. Among these, two scales received a Grade A recommendation for use in clinical or research practice: the Self-evaluation Negative Symptom (SNS), which assesses the five domains of NSs, and the Motivation And Pleasure Scale Self-report (MAP-SR), which evaluates anhedonia, avolition, and social withdrawal. **Conclusions**: The SNS and the MAP-SR are the only tools with sufficient psychometric properties, making them reliable for use in both research and clinical practice. Despite the development of self-assessment tools for NSs, their integration into research and clinical settings remains limited, highlighting the need for increased utilization to enhance the understanding and management of these symptoms.

## 1. Introduction

Schizophrenia is a complex psychiatric disorder with various symptoms, the most disabling of which are negative symptoms (NSs). NSs are categorized into five dimensions: anhedonia, avolition, blunted affect, social withdrawal, and alogia [1]. The early detection and continuous monitoring of NSs are crucial for developing optimal and personalized treatment strategies. NSs significantly impact the progression of the disease due to their high prevalence [2], their effect on daily life [3], and their resistance to treatments [4].

Several scales have been developed to assess NSs in patients with schizophrenia, most of which rely on observer ratings [5], and several papers have reported their psychometric properties [6,7,8]. These tools require clinical interviews with trained psychiatrists and are not suitable for evaluating patients’ subjective experiences. Despite their potential, self-assessment tools remain underused in clinical practice and research. This underutilization may stem from concerns about their psychometric robustness compared to observer-rated tools or a lack of familiarity among clinicians and researchers.

The integration of self-assessment scales for evaluating symptomatology has emerged as a promising approach for understanding and managing psychotic disorders. Patients’ self-assessments can provide insights into their own experiences that may not be detected by clinicians during interviews [9,10]. By placing patients at the center of their care, these tools can improve awareness and understanding of the illness, thereby increasing engagement in treatment and empowering patients [11]. Furthermore, self-assessment scales offer practical advantages for large-scale studies or routine clinical monitoring, where observer-rated tools may be less feasible due to time and resource constraints [12]. Additionally, self-assessments are particularly useful for the early detection of psychotic symptoms [13]. They also have potential utility in patients with treatment-resistant schizophrenia, although their use may be limited in cases of significant cognitive impairment or low functioning [14]. Although self-assessment scales provide essential insights into the patients’ subjective experience, they are susceptible to biases that can lead to both overdiagnosis and underdiagnosis. These biases can arise when patients engage in socially desirable responding, potentially underreporting or overreporting symptoms. A study comparing self-ratings with observer ratings highlighted this issue, with healthy individuals tending to overestimate symptoms, while patients may underreport them [15]. However, these limitations underscore the importance of integrating self-assessments with clinician-rated tools to ensure diagnostic accuracy and optimize treatment strategies. Such a combined approach not only enhances diagnostic accuracy but also allows for more effective treatment of NSs.

The European Psychiatric Association recommends combining hetero-evaluation tools, such as the Brief Negative Syndrome Scale (BNSS) [16] or the Clinical Assessment Interview for Negative Symptoms (CAINS) [17], with self-assessment scales, such as the Self-evaluation of Negative Symptoms (SNS) [18] or the Motivation And Pleasure-Self Report (MAP-SR) [19,20]. These recommendations emphasize the need for a comprehensive approach to assessing NSs that considers both clinician-rated and self-reported measures across the five relevant dimensions. Along these lines, new self-report tools have been developed, but paradoxically, they are underused in clinical practice and research. Some of these tools assess only a single dimension of NSs, which limits their clinical utility. While specialized tools targeting specific dimensions, such as motivation or anhedonia, are valuable for focused research or interventions [21], they do not provide a complete picture of NSs [22]. Given that NSs are complex and multifactorial, an effective evaluation tool must consider the interplay between multiple dimensions to guide clinical decision-making. Therefore, this review focuses on self-assessment scales that evaluate multiple domains of NS. The aim is to identify the most relevant tools for clinical practice and research by evaluating their psychometric properties to ensure a comprehensive assessment of NSs.

## 2. Materials and Methods

Data for this systematic review were collected in accordance with the Systematic Reviews and Meta-Analyses guidelines [PRISMA] (Supplementary Materials) [23]. This review was not pre-registered.

### 2.1. COSMIN Methodology

The Consensus-based Standards for the Selection of health Measurement Instruments (COSMIN) checklist was used to ensure a rigorous evaluation of the methodological quality of each self-assessment scale included in this review [24,25,26]. COSMIN methodology was chosen for its structured approach to assessing the psychometric quality of measurement tools. The COSMIN steps involved in this study were: conducting a comprehensive literature search, utilizing the Risk of Bias checklist, applying updated criteria for good measurement properties, implementing the GRADE approach to evaluate evidence quality, and developing evidence-based recommendations.

### 2.2. Search Method

A systematic electronic search was conducted in the MEDLINE and Web of Science databases. The following search query were used in both PubMed and Web of Sciences databases, incorporating the names of tools identified in a previous review [5]: For PubMed, the search included:

“Psychometric validation AND (self-report scale OR self-assessment scale OR Self-Evaluation of Deficit Syndrome OR SEDS OR Subjective Experience of Negative Symptoms OR SENS OR Motivation And Pleasure Scale Self-report OR MAP-SR OR Self-evaluation of Negative Symptoms OR SNS) AND (negative symptoms OR anhedonia OR avolition OR motivation OR social withdrawal OR blunted affect OR alogia) AND (psychotic disorders OR schizophrenia OR schizoaffective disorder OR first-episode psychosis OR ultra-high risk of psychosis OR FEP OR UHR)”. 

In Web of Science, the search query was: “Psychometric validation AND self-report scale AND negative symptoms AND schizophrenia”.

**Figure 1:**
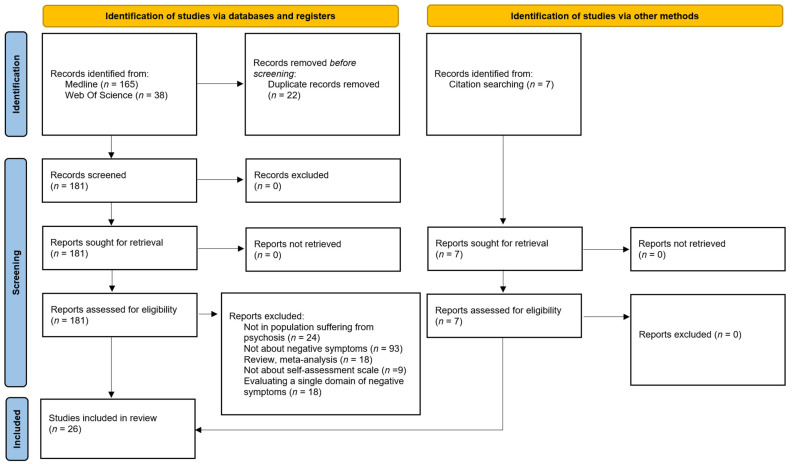
Flow-chart.

Inclusion criteria encompassed studies published in English or French from 1987 to November 2024 that evaluated the psychometric properties of self-assessment tools specifically developed to detect or assess NSs in psychotic disorders, including schizophrenia, schizoaffective disorder, first-episode psychosis, and ultra-high risk of psychosis. Exclusion criteria included studies on other disorders, meta-analyses or literature reviews, studies not reporting the psychometric characteristics of the clinical tools, studies not focusing on self-assessment scales or on NS, and tools focused specifically on only one domain of NSs (Figure 1).

Although meta-analyses and literature reviews were excluded from the electronic search, a manual search of their bibliographies was conducted to identify additional validation studies for self-assessment tools not initially included. Data were extracted and analyzed by one author (LM).

For each tool, details regarding the population (number, age, and gender), number of items, psychometric properties, countries of validation, and negative dimensions were documented.

### 2.3. Assessing the Risk of Bias and Updated Criteria for Good Measurement Properties

The COSMIN methodology [24,25,26] was applied to evaluate both the risk of bias and the measurement properties of the self-assessment tools for NS. 

In this review, we focused on the following psychometric properties: internal consistency (via Cronbach’s alpha, contingent on evidence of structural validity), test–retest reliability (evaluated using the intraclass correlation coefficient (ICC)), structural validity (analyzed through factor analysis), criterion validity (evaluated by Area Under Receiver Operating Characteristic Curve (AUROC) analysis), and hypothesis testing for construct validity based on convergent and discriminant validity (ensuring predefined hypotheses were tested). Finally, responsiveness was excluded due to the absence of a true gold standard measure for the self-assessment of negative symptoms, and cross-cultural validity/measurement invariance were not assessed, as they were addressed in only one study [27].

Initially, we utilized the COSMIN Risk of Bias Checklist, where each measurement property was rated on a four-point scale (“very good”, “adequate”, “doubtful”, “inadequate”, or “not applicable”). When several studies were identified assessing a psychometric property, the “worst score counts” rule was applied, meaning that the overall rating was the lowest score. The second phase assessed the measurement properties according to the updated COSMIN criteria, with each property rated as “sufficient” (+), “insufficient” (−), or “indeterminate” (?). The “indeterminate” rating was assigned when the data were insufficient for a conclusive assessment.

### 2.4. GRADE Approach

The Grading of Recommendations Assessment, Development, and Evaluation (GRADE) focuses on the confidence in the quality of evidence [24,25,26]. This approach evaluates evidence quality based on four factors: (1) risk of bias, assessing the methodological quality of studies; (2) inconsistency, referring to unexplained variations in results across studies; (3) imprecision, considering the total sample size of the available studies; and (4) indirectness, indicating whether the evidence comes from populations different from the one targeted in the review. Each factor allows for downgrading by one or more levels, and downgrades can be cumulative across the four criteria. The last criterion was not considered in the present study, as the studies were selected based on specific populations.

The process begins with the assumption that the result is of high quality. However, if the findings are deemed insufficient, the quality may be downgraded, resulting in the categorization of evidence quality as “high”, “moderate”, “low”, or “very low”. 

From the assessment of the “risk of bias”, only the studies that received a “+” or “−” rating based on the updated criteria for good measurement were considered for downgrading the confidence in the evidence. Downgrades were applied as follows:

- No downgrade: multiple studies of at least adequate quality, or a single study of very good quality available;
- −1 downgrade, serious risk of bias: multiple studies of doubtful quality available, or only one study of adequate quality;
- −2 downgrades, very serious risk of bias: multiple studies of inadequate quality, or only one study of doubtful quality available;
- −3 downgrades, extremely serious risk of bias: only one study of inadequate quality available.

Regarding “inconsistency”, the criteria for downgrading were established as follows: the rating on the scale remained unchanged if no inconsistency, or only minimal inconsistency with a valid explanation, was observed. Conversely, if minor inconsistency was found without any explanation, or if moderate to high inconsistency occurred with a valid justification, the rating on the scale was downgraded by one level (serious). In instances of moderate to high inconsistency without a satisfactory explanation, a downgrade of two levels (very serious) was applied.

For “imprecision”, the total sample size of all the studies was considered, with a downgrade of one level for sample sizes between 50 and 100, and a downgrade of two levels for sample sizes smaller than 50. 

### 2.5. Recommendations

Using the information from the previous section, COSMIN recommendations [24,25,26] for the level of evidence of the different self-assessment scales are formulated as follows:

(A) Sufficient content validity evidence (of any level) and at least low-quality evidence for adequate internal consistency.
(B) This category includes studies that do not qualify for either category A or C. It encompasses those with inadequate results, such as missing information on specific measurement properties, preventing classification into either category.
(C) High-quality evidence demonstrating an insufficient measurement property.

This classification framework serves as a guide for recommendations on the clinical use and application of these scales.

## 3. Results

Twenty-six articles related to five self-assessment scales were analyzed, covering several NS domains. Table 1 provides the following information about the scales. The COSMIN quality criteria for good measurement, the risk of bias, the Grade evaluation, and recommendation are outlined in Table 2 for self-assessment scales with multiple validation studies. For scales with a single validation study, detailed results are provided exclusively in the text.

### 3.1. The Subjective Experience of Negative Symptoms (SENS)

The Subjective Experience of Negative Symptoms (SENS) [28] assesses “affective flattening”, “alogia”, “avolition-apathy”, “anhedonia-asociality”, and “attention” through 21 items. 

#### 3.1.1. Risk of Bias and Criteria for Good Measurement Properties

Only one study [28] evaluated the psychometric properties of the SENS, focusing solely on internal consistency and reliability. The risk of bias regarding internal consistency was rated as “doubtful” due to a lack of information regarding the scale’s structural validity, which led to the measurement properties being rated as “indeterminate”. 

Regarding reliability, the risk of bias was assessed as “adequate”, but the overall evaluation of the measurement properties was “negative” due to the ICC being under 0.70.

#### 3.1.2. GRADE Evaluation

Insufficient findings led to a downgrade of −1. Since there was only one study of adequate quality assessing reliability, this resulted in a serious risk of bias and an additional downgrade of −1.

The criterion for inconsistency was not applicable, as there was only one validation study. 

For imprecision, as the sample size was *n* = 52, an additional downgrade of −1 was applied, resulting in a “very low” grade for the SENS.

Thus, the overall quality of the evidence for the SENS scale was considered “very low”, and the level of recommendation was C.

### 3.2. The Clinical Assessment Interview for Negative Symptoms-Self-Report (CAINS-SR)

The Clinical Assessment Interview for Negative Symptoms – Self-Report (CAINS-SR) [29] is a 30-item self-report measure based on the CAINS interview version, which evaluates the five domains of NSs.

#### 3.2.1. Risk of Bias and Criteria for Good Measurement Properties

Only one study [29] examined the psychometric properties of the CAINS-SR, focusing on internal consistency and testing hypotheses for construct convergent and discriminant validity. 

The risk of bias for internal consistency was rated as “doubtful” due to limited information on the scale’s structural validity, leading to an “indeterminate” assessment of the measurement properties. 

For hypothesis testing of both convergent and discriminant validity, the risk of bias was considered “adequate”, and the measurement properties were rated “positive”. The “experience” and “motivation” dimensions of the CAINS-SR were compared with the corresponding dimensions rated by clinicians on the CAINS [17] for convergent validity, as well as with the “positive”, “agitation/mania”, and “depression/anxiety” dimensions of the Brief Psychiatric Rating Scale (BPRS) [50] for discriminant validity.

#### 3.2.2. GRADE Evaluation

As the findings were insufficient, a downgrade of −1 was applied. Since there was only one study of adequate quality, this led to a serious risk of bias and an additional downgrade of −1. 

The criterion for inconsistency was not applicable, as only one validation study was available.

For imprecision, the sample size was *n* = 73, which led to an additional downgrade of −1. 

Thus, the overall quality of the evidence for the CAINS-SR scale was considered “very low” and the level of recommendation was C.

### 3.3. The Motivation and Pleasure Scale Self-Report (MAP-SR)

The Motivation and Pleasure Self-Report scale (MAP-SR) [19] is derived from the CAINSs-SR and comprises three negative dimensions: anhedonia, avolition, and social withdrawal. This 15-item scale assesses consummatory and anticipatory pleasure related to social, recreational, and work domains, as well as motivation related to family and activities.

#### 3.3.1. Risk of Bias and Criteria for Good Measurement Properties

Structural validity was assessed in two [31,33] of the seven validation studies of the MAP-SR [19,30,31,32,33,34,35]. One study [31] received a “very good” rating for risk of bias and a “positive” rating for measurement properties, indicating an adequate fit for a four-factor model. In contrast, the other study [33] received a “doubtful” rating for risk of bias due to a sample size of less than 100 and a “negative” rating for measurement properties, as some factor loadings did not reach the 0.30 threshold.

All seven validation studies of the MAP-SR assessed internal consistency [19,30,31,32,33,34,35]; however, only two studies [31,33] evaluated structural validity, receiving a “very good” rating for risk of bias and a “positive” rating for measurement properties. The remaining studies [19,30,32,34,35] did not include factor analysis, resulting in “doubtful” ratings and “indeterminate” outcomes due to insufficient evidence supporting adequate structural validity for the MAP-SR.

Only two studies assessed the reliability of the MAP-SR [31,33], both receiving an “adequate” rating for risk of bias. One study [31] achieved a “positive” rating for measurement properties, while the other [33] received a “negative” rating due to its failure to meet the 0.7 threshold for correlation coefficients across all subscales.

All seven MAP-SR validation studies [19,30,31,32,33,34,35] conducted hypothesis testing for both convergent and discriminant validity. Five studies received an “adequate” rating [19,30,31,34,35] for risk of bias, while two were rated ‘very good’ [32,33]. Overall, the studies achieved a “positive” rating for the measurement properties of convergent validity [19,30,31,32,34,35], except for one study [33], which received a “negative” rating due to correlation coefficients below 0.5. Convergent validity was established by comparing the MAP-SR with various scales, including the BPRS negative subscore [50], the Positive and Negative Syndrome Scale (PANSS) negative subscore [51], the Scale for the Assessment of Negative Symptoms (SANS) [52], CAINS [17], SNS [18], and the Temporal Experience of Pleasure Scale (TEPS) [53]. For discriminant validity, four studies [19,30,31,32] were rated as “positive” for comparing the MAP-SR against scales for depression, positive symptoms, quality of life, and medication side effects. However, three studies [33,34,35] received an “indeterminate” rating. Two of these studies assessed discriminant validity solely through correlations with depression scales, which yielded moderate correlations that were insufficient to confirm strong discriminant validity [34,35]. Additionally, the authors of one study did not clearly state specific hypotheses regarding discriminant validity [33].

#### 3.3.2. GRADE Evaluation

There were either multiple studies of adequate quality or a single study of very good quality available for structural validity, reliability, internal consistency, and hypothesis testing for both construct convergent and discriminant validity. Consequently, no downgrading was applied. 

Due to the inconsistency in the results of the reliability studies, a −1 downgrade was applied.

For imprecision, with the overall sample size for the MAP-SR being *n* = 676, no downgrade was applied.

Thus, the overall quality of the evidence for the MAP-SR scale was considered “moderate” and the level of recommendation was A.

### 3.4. The Self Evaluation of Negative Symptoms (SNS)

The Self-evaluation of Negative Symptoms (SNS) [18] provides an assessment of all five domains of NSs using 20 items, scored from 0 (no symptoms) to 40 (severe negative symptoms), within a brief timeframe. The SNS is available in 27 languages (see https://sns-dollfus.com, accessed on 10 November 10 2024), ensuring global accessibility. 

#### 3.4.1. Risk of Bias and Criteria for Good Measurement Properties

Among the seventeen validation studies of the SNS [9,14,18,36,37,38,39,40,41,42,43,44,45,46,47,48,49], nine [14,18,38,41,42,45,46,47,48] specifically assessed its structural validity. Of these, five studies [14,18,42,45,47] were rated as “inadequate” in terms of risk of bias, primarily due to an insufficient number of participants for factor analysis (fewer than five times the number of items on the scale). One study [38] received a “doubtful” rating due to the inappropriate use of principal component analysis. In contrast, three studies [41,46,48] received a “very good” rating for structural validity. Regarding measurement properties, eight studies were rated as “positive” [18,38,41,42,45,46,47,48], demonstrating a good fit for either a two-factor model [18,42,47] or a five-factor model [38,41,45,46,48], while only one study [14] was rated as “indeterminate”.

Twelve studies [14,18,36,37,38,40,42,43,44,45,47,48] assessed the internal consistency of the SNS. Among these, one study [48] was rated as having a “very good” risk of bias, while eleven studies [14,18,36,37,38,40,42,43,44,45,47] were deemed “doubtful”. The primary reasons for this rating included the absence of Cronbach’s alpha calculations for all subscales and insufficient information regarding the scale’s structure, either due to a lack of factor analysis or the use of an inadequate model. In terms of measurement properties, four studies [14,18,42,47] received “positive” ratings, whereas two studies [45,48] were rated “negatively” because their Cronbach’s alpha coefficients fell below the accepted threshold of 0.7. Additionally, six studies [36,37,38,40,43,44] received “indeterminate” ratings, as they failed to meet the criteria for providing at least minimal evidence of sufficient structural validity or lacked Cronbach’s alpha calculations for all SNS subscales.

The reliability of the SNS was assessed in four studies [14,18,36,38]. Two studies [14,18] received “very good” ratings for risk of bias and “positive” ratings for measurement properties, as they reported ICC values exceeding 0.7. In contrast, one study [38] was rated as having an “adequate” risk of bias but received a “negative” rating for measurement properties, with all ICC scores falling below the 0.7 threshold. Finally, another study [36] was rated as “inadequate” for risk of bias due to its reliance on a *t*-test for reliability assessment, rather than the recommended methods. Consequently, this study also received an “indeterminate” rating for its measurement properties.

Five studies with a “very good” risk of bias rating evaluated criterion validity [9,39,47,48,49]. All of these studies received a “positive” rating for measurement properties, with area under the curve (AUC) values greater than 0.7. The results demonstrate that the SNS is able to identify thresholds for the severity of NSs among patients at ultra-high risk of psychosis [49], experiencing their first episode of psychosis [39], diagnosed with schizophrenia [9,48], or presenting with a deficit subtype [47].

Finally, hypothesis testing for construct convergent validity was conducted across thirteen validation studies for the SNS [14,18,36,37,38,40,42,43,44,45,46,47,48]. The risk of bias was rated as “very good” for eight studies [14,18,38,40,43,44,46,48] and “adequate” for five studies [36,37,42,45,47]. Regarding measurement properties, twelve studies received a “positive” rating [18,36,37,38,40,42,43,44,45,46,47,48]. Only one study [14] received a “negative” rating because the correlations failed to reach the threshold of 0.5 with other measures of NS. In the various studies, the SNS was compared with the Scale for the Assessment of Negative Symptoms (SANS) [52], the BPRS negative subscore [50], the PANSS negative subscore [51], the Clinical Global Impression (CGI) negative subscore [54], the BNSS [16], the Negative Symptom Assessment 16 (NSA-16) [55] and the CAINS [17] for convergent validity. In evaluating hypothesis testing for construct discriminant validity, nine studies [14,18,36,38,40,42,44,45,46] were included. Among them, three studies [14,18,46] received a “very good” rating for risk of bias, while four studies [36,38,42,45] were rated as “adequate”. All of them [14,18,36,38,42,45,46] obtained a “positive” rating for measurement properties. These studies effectively compared the SNS with scales assessing depression, parkinsonism, and positive symptoms to determine discriminant validity. Conversely, two studies [40,44] were rated as ’doubtful’ for risk of bias and received an ’indeterminate’ rating for measurement properties due to their exclusive use of a quality of life scale, which is insufficient for determining discriminant validity.

#### 3.4.2. GRADE Evaluation

Since there were several studies of at least adequate quality, or one study of very good quality available for each criterion, the scale was not downgraded in terms of its risk of bias.

Concerning inconsistency, only internal consistency and reliability showed conflicting results, which led to a downgrade of −1. Inconsistency was not considered in hypothesis testing for construct convergent validity, as eleven studies reported “positive” outcomes and only one received a “negative” rating. 

For imprecision, with the overall sample size for the SNS being *n* = 1835, no downgrade was applied.

Thus, the overall quality of the evidence for the SNS scale was considered “moderate”, and the level of recommendation was A.

### 3.5. The Negative Symptoms Inventory Self-Report (NSI-SR)

The Negative Symptoms Inventory Self-Report (NSI-SR) [27] evaluates three domains of negative symptomatology (avolition, anhedonia, and social withdrawal) with eleven items. This study was conducted with students, individuals at ultra-high risk of psychosis, and those with schizophrenia.

#### 3.5.1. Risk of Bias and Criteria for Good Measurement Properties

Only one study [27] evaluated the psychometric properties of the NSI-SR, focusing on structural validity, internal consistency, reliability, and hypothesis testing for construct convergent and discriminant validity.

The risk of bias was assessed as “very good” for structural validity, supported by a confirmatory factor analysis conducted with an adequate number of subjects. Measurement properties were rated as “positive”, supported by favorable fit indices from the confirmatory factor analysis, which identified a three-factor model.

The internal consistency of the NSI-SR was rated as “very good” for risk of bias and received a “positive” rating for measurement properties, with Cronbach’s alpha exceeding 0.7 for all three factors of the scale.

The risk of bias for reliability was rated as “adequate”, and the measurement properties were rated as “negative” due to correlation scores below 0.7.

Finally, the risk of bias for hypothesis testing of construct convergent and discriminant validity was rated as “very good”. Regarding measurement properties, convergent validity, assessed using BNSS scores [16], received a “negative” rating due to low correlation levels. In contrast, discriminant validity was also rated as “negative” because of positive correlations with scores from depression and hallucination scales. 

#### 3.5.2. GRADE Evaluation

Since there was only one study [27] of adequate quality assessing reliability, this resulted in a “serious” risk of bias and a downgrade of −1. 

The criterion for inconsistency was not applicable, as there was only one validation study available with determinable results.

For imprecision, since the sample size was 62, an additional downgrade of −1 was applied.

Thus, the overall quality of the evidence for the NSI-SR scale was considered “low”, and the level of recommendation was B.

## 4. Discussion

This review highlights various self-assessment instruments specifically designed for the evaluation of several dimensions of NSs in patients with schizophrenia, schizoaffective disorder, a first episode of psychosis, or those at ultra-high risk of psychosis. We have rigorously evaluated their psychometric properties to facilitate their application in clinical practice and research. In the following discussion, we will delve into the strengths, weaknesses, and recommendations for the different tools. 

Among the scales that cover several dimensions of NSs, three (SENS [28], CAINS-SR [29], and SNS [18]) assess five domains of NSs, while two others assess three domains. Although the SENS [28] provides comprehensive coverage, it has significant limitations. One limitation concerns the assessment of anhedonia, as it fails to account for its two dimensions: anticipatory and consummatory pleasure. Additionally, the inclusion of ’attention’ has been a subject of debate, as it may be more closely related to cognitive deficits rather than NSs [5]. The COSMIN evaluation further highlights weaknesses, with only one validation study available [28]. This study reported negative ratings for test–retest reliability, indeterminate results for internal consistency, and the absence of structural validity testing or adequate hypothesis testing for construct validity (both convergent and discriminant). As a result, the scale has a recommendation rating of C.

Similarly, the CAINS-SR [29] offers a comprehensive evaluation of all domains of negative symptomatology. However, its psychometric properties have been examined in only one study [29]. While this study reported promising results in hypothesis testing of construct convergent and discriminant validity, there is insufficient information regarding other validation domains. Given the limited evidence available, it has received a recommendation rating of C.

In contrast, the SNS [18] demonstrates robust psychometric properties and effectively assesses all five domains of negative symptomatology, including both anticipatory and consummatory anhedonia. A notable strength of this scale is its ability to identify thresholds in the severity of NSs among patients with schizophrenia [9], first-episode psychosis [39], healthy adolescents [56], and students [49]. This highlights the SNS’s capability to detect NSs across diverse populations. Numerous studies [9,14,18,36,37,38,39,40,41,42,43,44,45,46,47,48,49], including several of high quality [46,48], have evaluated the psychometric properties of the SNS, demonstrating strong results across all measurement domains. These findings support a grade A recommendation for using the SNS in both clinical and research settings for the schizophrenia population.

Other tools, such as the MAP-SR [19] and NSI-SR [27] offer targeted assessments of key NS domains, including asociality, avolition, and anhedonia. The MAP-SR [19] provides a detailed evaluation of anticipatory and consummatory anhedonia across various contexts (social, recreational, and professional) and assesses motivation in family relationships and activities [19]. This specificity enhances the understanding of these critical dimensions of NS, which are often challenging to evaluate accurately. Several high-quality studies [31,33] have evaluated the MAP-SR, confirming its strong psychometric properties and supporting a grade A recommendation for its use in routine practice and research in the schizophrenia population.

Lastly, the NSI-SR [27] assesses both consummatory and anticipatory pleasure, facilitating a focused evaluation of anhedonia. It also measures social withdrawal and engagement in social activities, effectively highlighting challenges in social interactions. Additionally, it addresses avolition through items related to motivation and the initiation of activities. A notable strength of the NSI-SR is its validation across diverse populations, including students, individuals at ultra-high risk of psychosis, and those with schizophrenia, enhancing its capacity to detect NSs in various contexts [27]. However, the psychometric properties of the NSI-SR have been evaluated in only one study [27]. While this research reported promising findings regarding structural validity and internal consistency, other evaluations highlighted poor performance in test–retest reliability and hypothesis testing for construct convergent and discriminant validity. Due to the limited number of validation studies, the NSI-SR has been assigned a recommendation rating of B.

In summary, while some evidence supporting these self-assessment tools is of low or moderate quality, these instruments remain valuable for both clinical practice and research. The low-quality evidence often reflects a lack of validation studies rather than inherent weaknesses in the tools themselves, except for the SENS [28] scale, which does not adequately evaluate NSs, notably anhedonia. Among these tools, the SNS [18] and MAP-SR [19] stand out with grade A recommendations, reflecting their robust psychometric properties. The SNS excels in covering the five domains of negative symptomatology, while the MAP-SR is particularly effective for targeted evaluations of anhedonia, avolition, and social withdrawal. These strengths position them as reliable and clinically relevant instruments. In contrast, the CAINS-SR [29] and NSI-SR [27], though promising, require further research to solidify their psychometric foundations. Despite this limitation, they offer valuable perspectives and hold potential to complement observer-rated measures, particularly by capturing patients’ subjective experiences, which are often overlooked in clinical assessments.

In addition to these self-assessment tools, other instruments focus on single dimensions of NSs, limiting their clinical utility in offering a comprehensive view of the patient’s condition. For example, the Temporal Experience of Pleasure Scale (TEPS) [53] specifically targets anhedonia, while the BIRT Motivation Questionnaire (BMQ-S) [57] measures motivational deficits. These focused tools are valuable in research and specific clinical contexts, such as cognitive behavioral therapy, which has been shown to be effective in addressing symptoms related to the avolition–apathy dimension of NSs [21]. Assessing these particular aspects of NSs can be highly relevant, as it allows clinicians to guide targeted interventions—such as behavioral activation strategies for avolition or activities designed to foster enjoyment [21]. However, their narrow focus limits their ability to provide a comprehensive understanding of the multiple dimensions of NSs [22], potentially overlooking the synergistic effects between NS domains and their cumulative impact on patient functioning [58]. Therefore, without the integration of multi-dimensional scales, single-dimension tools may fail to capture the full complexity of NSs, potentially leading to incomplete assessments, missed treatment effects, and suboptimal care planning [59]. In contrast, multi-dimensional tools offer a more holistic assessment by examining the interplay between different domains of NSs and their combined effect on daily functioning [60]. This is crucial for identifying distinct NS profiles, which can help inform personalized therapeutic strategies. Moreover, in clinical trials assessing the effects of antipsychotics on secondary NSs and experimental treatments targeting NSs, such as neurostimulation interventions, multi-dimensional tools are indispensable for capturing subtle, multi-faceted changes across various dimensions of NSs [1]. These tools also play a pivotal role in research, as they enable a broader dataset to correlate clinical symptoms with neurobiological markers, enhancing our understanding of NS mechanisms and informing the development of future treatments.

In spite of the European Association’s recommendation [20], the use of self-assessment scales remains limited, even though robust tools are available. While Weigel et al. [7] assessed the psychometric properties of hetero-evaluation tools for NSs, including the SANS [52], High Royds Evaluation of Negativity Scale (HEN) [61], and Negative Symptom Assessment-16 (NSA-16) [55], concluding that these tools are unsuitable for clinical practice, other studies have highlighted the strong psychometric properties of newer instruments such as the BNSS and the CAINS [6,8]. Specifically, Wehr et al. [6] highlighted the robust measurement properties of the CAINS in terms of structural validity, internal consistency, and reliability, while Weigel et al. [8] underscored the strong structural validity, internal consistency, and interrater reliability of the BNSS across various contexts. In the same vein, our study focuses on self-assessment tools and underscores the strong psychometric properties of the SNS [18] and the MAP-SR [19], which provide alternatives for clinical and research applications. These self-assessment scales, with their robust validation, could complement traditional observer-rated evaluations, contributing to a more comprehensive approach to NS assessment, as recommended by the European Psychiatric Association [20]. The incorporation of self-assessment scales into clinical routines is crucial, as they can significantly enhance our understanding of patients’ experiences [9,10] and inform the development of personalized treatment strategies. Furthermore, incorporating self-assessments into emerging technologies, such as digital platforms and ecological momentary assessments, could facilitate real-time data collection, enabling the capture of fluctuations in NSs and their interactions with motivational factors in patients’ daily lives [62]. By leveraging digital platforms, clinicians can gain deeper insights into patients’ experiences, thereby enhancing the accuracy of self-reports and fostering more personalized approaches to care [62,63]. 

One important aspect to consider in the use of these self-assessment scales is their availability in different cultures and languages, as well as the practical barriers to their implementation in diverse clinical settings. Many of these scales were developed and validated in specific cultural and linguistic contexts, limiting their use in populations that do not have access to an adapted version. Therefore, applying a rigorous translation and cultural adaptation process is essential to ensure that these tools are appropriate and relevant in different contexts. This process must take into account linguistic and cultural nuances to avoid assessment biases and ensure reliable use of the instruments [64]. In low-resource settings, where access to these tools may be limited, these challenges may be even more pronounced. Therefore, while these scales show promising potential in research, efforts should be made to enhance their accessibility to a broader range of populations and to support their adaptation in order to maximize their effectiveness across diverse clinical environments.

Despite employing a recommended methodology (COSMIN), our study has several potential limitations. First, data extraction and analysis were conducted by a single reviewer, which may introduce bias or lead to oversight in the interpretation of results. To mitigate this concern, we used the COSMIN framework, a robust tool for evaluating the psychometric properties of assessment instruments. Second, we did not perform a meta-analysis to confirm our findings due to the diverse nature of the data, which prevented us from quantitatively summarizing the results. Such an analysis would have provided a more precise estimate of the psychometric properties across studies, potentially revealing overall effect sizes and the variability in results. Finally, no protocol was established prior to conducting this review, and the process was not registered. However, the review was carried out following standard systematic approaches, with clear inclusion and exclusion criteria, ensuring transparency and minimizing bias. The absence of a formal registration does not affect the reliability or validity of the findings, as the methodology remained consistent with established practices in the field.

## 5. Conclusions

In conclusion, this review emphasizes that, despite the underuse of self-assessment scales for NSs in schizophrenia, several scales are available for clinicians and researchers. Among these, two instruments receive a Grade A recommendation for clinical or research practice: the SNS [18] for assessing the five domains of NSs and the MAP-SR [19] for evaluating three negative dimensions: anhedonia, avolition, and social withdrawal. 

## 6. Future Directions

Future research should prioritize enhancing the psychometric properties of existing self-assessment scales for measuring NSs in schizophrenia. This should include longitudinal studies to establish test–retest reliability and investigate how demographic and clinical factors affect scale performance. Furthermore, validating these tools in diverse clinical settings, including both outpatient programs and inpatient units, is crucial. Ultimately, this will help integrate these scales into routine clinical practice and improve outcomes for individuals with schizophrenia.

## Figures and Tables

**Table 1:** General characteristics of self-assessment scales with more than 2 negative symptom domains.

Scale	Population	Mean Age (SD)	Gender (Female %)	Countries Validation	Negative Symptoms Domains	Number of Items	Duration
**Subjective Experience of Negative Symptoms (SENS)** Selten et al., 1993 [28]	52 patients with schizophrenia	39.5 (10.8)	34.6	The Netherlands	Anhedonia/asociality Avolition/apathy Affective flattening Alogia Attention	21	-
**Clinical Assessment Interview for Negative Symptoms Self-Report (CAINS-SR)** Park et al., 2020 [29]	41 patients with schizophrenia 32 patients with schizo-affective disorders	47.1 (8.36)	36.2	North America	Anhedonia Avolition Alogia Blunted affect Asociality	30	-
**Motivation And Pleasure Scale** **Self-Report (MAP-SR)** Llerena et al., 2013 [19] Engel et al., 2016 [30] Wang et al., 2020 [31] Kim et al., 2016 [32] Richter et al., 2019 [33] Garcia-Portilla et al., 2021 [34] Cernvall et al., 2024 [35]	37 patients with schizophrenia or schizo-affective disorders 50 patients with schizophrenia or schizo-affective disorders 150 patients with schizophrenia 139 patients with schizophrenia 93 patients with schizophrenia or schizo-affective disorder 174 patients with schizophrenia 33 patients with schizophrenia spectrum disorders	50.16 (5.12) 35.70 (10.36) 46.47 (8.37) 38.9 (11.1) 38.99 (10.99) 36.7 (12.2) 40.0 (11.0)	35.1 44 51.3 45.3 33.3 37.4 36	North America Germany China Korea German Spain Sweden	Anhedonia Avolition Social withdrawal	15	-
**Self-evaluation of Negative Symptoms (SNS)** Dollfus et al., 2016 [18] Hervochon et al., 2018 [36] Wojciak et al., 2019 [37] Dollfus et al., 2019 [9] Hajj et al., 2019 [38] Mallet et al., 2020 [39] Garcia-Alvarez et al., 2020 [40] Goldring et al., 2020 [14] Tam et al., 2021 [41] Montvidas et al., 2021 [42] Mazhari et al., 2021 [43] Garcia-Alvarez et al., 2022 [44] Polat et al., 2022 [45] Dollfus et al., 2022 [46] Samochowiec et al., 2023 [47] Chen et al., 2023 [48] Métivier et al., 2024 [49]	23 patients with schizophrenia 26 patients with schizo-affective disorders 60 patients with schizophrenia or schizo-affective disorders 40 patients with schizophrenia 109 patients with schizophrenia or schizo-affective disorders 99 healthy controls 206 patients with schizophrenia 29 patients with schizophrenia 22 patients with depressive disorder 59 healthy controls 104 patients with schizophrenia 50 patients with resistant schizophrenia 204 patients with schizophrenia 67 patients with schizophrenia 50 patients with schizophrenia 104 patients with schizophrenia 75 patients with schizophrenia 245 patients with schizophrenia 82 patients with schizophrenia 200 patients with schizophrenia 367 students with ultra-high risk of psychosis or major depressive disorders 1761 healthy students	36.7 (11.6) 40.6 (10.7) 44.0 (13.0) 38.9 (11.3) 28.8 (13.2) 52.68 (12.0) 19.4 (3.0) 18.0 (2.0) 20.4 (2.8) 40.1 (13.9) 43.8 (11.19) 49.36 (10.23) 41.51 (13.76) 39.5 (11.1) 40.1 (13.9) 21.91 (5.44) 37.4 (11.3) NR 35.2 (3.9) NR NR	20.4 20 50 NR NR 56.8 36.8 59.1 62.7 35.6 12 51.47 64.2 32 35.6 36 37 NR 53.5 NR NR	27 languages (www.sns-dollfus.com/fr, accessed on 10 November 2024) France France Poland France Lebanon France Spain North America Hong-Kong and China Lithuania Iran Spain Turkey European countries Poland China France	Anhedonia Avolition Alogia Reduced emotional range Social withdrawal	20	5 min
**Negative Symptoms Inventory Self-Report (NSI-SR)** Raugh et al., 2023 [27]	32 patients with schizophrenia or schizo-affective disorders 25 patients with clinical high-risk of psychosis	40.12 (13.25) 41.32 (9.43)	75 83.9	North America	Anhedonia Avolition Social withdrawal	11	Few minutes

SD: standard deviation; NR: not reported.

**Table 2:** COSMIN criteria of self-assessment scales with more than 2 negative symptom domains.

**MAP-SR** [19,30,31,32,33,34,35]		
**Measurement Property** **(No, of Study Assessing Measurement Property)**	**Risk of Bias**	**Update Criteria of Good Measurement**
**Structural validity (*n* = 2)** [31,33]	Very good (*n* = 1) [31] Doubtful (*n* = 1) [33]	Positive (*n* = 1) [31] Negative (*n* = 1) [33]
**Internal consistency (*n* = 7)** [19,30,31,32,33,34,35]	Very good (*n* = 2) [31,33] Doubtful (*n* = 5) [19,30,32,34,35]	Positive (*n* = 2) [31,33] Indeterminate (*n* = 5) [19,30,32,34,35]
**Reliability (*n* = 2)** [31,33]	Adequate (*n* = 2) [31,33]	Positive (*n* = 1) [31] Negative (*n* = 1) [33]
**Criterion validity (*n* = 0)**	/	/
**Hypotheses testing convergent validity (*n* = 7)** [19,30,31,32,33,34,35]	Very good (*n* = 2) [32,33] Adequate (*n* = 5) [19,30,31,34,35]	Positive (*n* = 6) [19,30,31,32,34,35] Negative (*n* = 1) [33]
**Hypotheses testing discriminant validity (*n* = 7)** [19,30,31,32,33,34,35]	Very good (*n* = 2) [32,33] Adequate (*n* = 5) [19,30,31,34,35]	Positive (*n* = 4) [19,30,31,32] Indeterminate (*n* = 3) [33,34,35]
**Quality of evidence (GRADE)**	Moderate	
**Recommendation**	A	
**SNS** [9,14,18,36,37,38,39,40,41,42,43,44,45,46,47,48,49]		
**Measurement property** **(no, of study assessing measurement property)**	**Risk of bias**	**Update criteria of good measurement**
**Structural validity (*n* = 9)** [14,18,38,41,42,45,46,47,48]	Very good (*n* = 3) [41,46,48] Doubtful (*n* = 1) [38] Inadequate (*n* = 5) [14,18,42,45,47]	Positive (*n* = 8) [18,38,41,42,45,46,47,48] Indeterminate (*n* = 1) [14]
**Internal consistency (*n* = 12)** [14,18,36,37,38,40,42,43,44,45,47,48]	Very good (*n* = 1) [48] Doubtful (*n* = 11) [14,18,36,37,38,40,42,43,44,45,47]	Positive (*n* = 4) [14,18,42,47] Negative (*n* = 2) [45,48] Indeterminate (*n* = 6) [36,37,38,40,43,44]
**Reliability (*n* = 4)** [14,18,36,38]	Very good (*n* = 2) [14,18] Adequate (*n* = 1) [38] Inadequate (*n* = 1) [36]	Positive (*n* = 2) [14,18] Negative (*n* = 1) [38] Indeterminate (*n* = 1) [36]
**Criterion validity (*n* = 5)** [9,39,47,48,49]	Very good (*n* = 5) [9,39,47,48,49]	Positive (*n* = 5) [9,39,47,48,49]
**Hypotheses testing for convergent validity (*n* = 13)** [14,18,36,37,38,40,42,43,44,45,46,47,48]	Very good (*n* = 8) [14,18,38,40,43,44,46,48] Adequate (*n* = 5) [36,37,42,45,47]	Positive (*n* = 12) [18,36,37,38,40,42,43,44,45,46,47,48] Negative (*n* = 1) [14]
**Hypotheses testing for discriminant validity (*n* = 9)** [14,18,36,38,40,42,44,45,46]	Very good (*n* = 3) [14,18,46] Adequate (*n* = 4) [36,38,42,45] Doubtful (*n* = 2) [40,44]	Positive (*n* = 7) [14,18,36,38,42,45,46] Indeterminate (*n* = 2) [40,44]
**Quality of evidence (GRADE)**	Moderate	
**Recommendation**	A	

MAP-SR: motivation and pleasure scale self-report; negative symptoms inventory self-report (NSI-SR); SNS: self-evaluation of negative symptoms.

## Supplementary Materials

The following supporting information can be downloaded at: https://www.mdpi.com/article/10.3390/brainsci15020148/s1, Table S1: PRISMA 2020 Checklist. Ref. [65] is cited in Supplementary Materials.

## References

[1] Kirkpatrick B., Fenton W.S., Carpenter W.T., Marder S.R. (2006). The NIMH-MATRICS Consensus Statement on Negative Symptoms. Schizophr. Bull..

[2] Sicras-Mainar A., Maurino J., Ruiz-Beato E., Navarro-Artieda R. (2014). Impact of Negative Symptoms on Healthcare Resource Utilization and Associated Costs in Adult Outpatients with Schizophrenia: A Population-Based Study. BMC Psychiatry.

[3] Montvidas J., Adomaitienė V., Leskauskas D., Dollfus S. (2021). Health-Related Quality of Life Prediction Using the Self-Evaluation of Negative Symptoms Scale (SNS) in Patients with Schizophrenia. Nord. J. Psychiatry.

[4] Stahl S.M., Buckley P.F. (2007). Negative Symptoms of Schizophrenia: A Problem That Will Not Go Away. Acta Psychiatr. Scand..

[5] Lincoln T.M., Dollfus S., Lyne J. (2017). Current Developments and Challenges in the Assessment of Negative Symptoms. Schizophr. Res..

[6] Wehr S., Weigel L., Davis J., Galderisi S., Mucci A., Leucht S. (2024). Clinical Assessment Interview for Negative Symptoms (CAINS): A Systematic Review of Measurement Properties. Schizophr. Bull..

[7] Weigel L., Wehr S., Galderisi S., Mucci A., Davis J.M., Leucht S. (2024). Clinician-Reported Negative Symptom Scales: A Systematic Review of Measurement Properties. Schizophr. Bull..

[8] Weigel L., Wehr S., Galderisi S., Mucci A., Davis J., Giordano G.M., Leucht S. (2023). The Brief Negative Symptom Scale (BNSS): A Systematic Review of Measurement Properties. Schizophrenia.

[9] Dollfus S., Delouche C., Hervochon C., Mach C., Bourgeois V., Rotharmel M., Tréhout M., Vandevelde A., Guillin O., Morello R. (2019). Specificity and Sensitivity of the Self-Assessment of Negative Symptoms (SNS) in Patients with Schizophrenia. Schizophr. Res..

[10] Lindström E., Lewander T., Malm U., Malt U.F., Lublin H., Ahlfors U.G. (2001). Patient-Rated versus Clinician-Rated Side Effects of Drug Treatment in Schizophrenia. Clinical Validation of a Self-Rating Version of the UKU Side Effect Rating Scale (UKU-SERS-Pat). Nord. J. Psychiatry.

[11] Eisen S.V., Dickey B., Sederer L.I. (2000). A Self-Report Symptom and Problem Rating Scale to Increase Inpatients’ Involvement in Treatment. Psychiatr. Serv..

[12] Mach C., Dollfus S. (2016). Scale for assessing negative symptoms in schizophrenia: A systematic review. Encephale.

[13] Niv N., Cohen A.N., Mintz J., Ventura J., Young A.S. (2007). The Validity of Using Patient Self-Report to Assess Psychotic Symptoms in Schizophrenia. Schizophr. Res..

[14] Goldring A., Borne S., Hefner A., Thanju A., Khan A., Lindenmayer J.-P. (2020). The Psychometric Properties of the Self-Evaluation of Negative Symptoms Scale (SNS) in Treatment-Resistant Schizophrenia (TRS). Schizophr. Res..

[15] Engel M., Lincoln T.M. (2017). Concordance of Self- and Observer-Rated Motivation and Pleasure in Patients with Negative Symptoms and Healthy Controls. Psychiatry Res..

[16] Kirkpatrick B., Strauss G.P., Nguyen L., Fischer B.A., Daniel D.G., Cienfuegos A., Marder S.R. (2011). The Brief Negative Symptom Scale: Psychometric Properties. Schizophr. Bull..

[17] Kring A.M., Gur R.E., Blanchard J.J., Horan W.P., Reise S.P. (2013). The Clinical Assessment Interview for Negative Symptoms (CAINS): Final Development and Validation. Am. J. Psychiatry.

[18] Dollfus S., Mach C., Morello R. (2016). Self-Evaluation of Negative Symptoms: A Novel Tool to Assess Negative Symptoms. Schizophr. Bull..

[19] Llerena K., Park S.G., McCarthy J.M., Couture S.M., Bennett M.E., Blanchard J.J. (2013). The Motivation and Pleasure Scale-Self-Report (MAP-SR): Reliability and Validity of a Self-Report Measure of Negative Symptoms. Compr. Psychiatry.

[20] Galderisi S., Mucci A., Dollfus S., Nordentoft M., Falkai P., Kaiser S., Giordano G.M., Vandevelde A., Nielsen M.Ø., Glenthøj L.B. (2021). EPA Guidance on Assessment of Negative Symptoms in Schizophrenia. Eur. Psychiatry.

[21] Schormann A.L.A., Pillny M., Haß K., Lincoln T.M. (2023). “Goals in Focus”-a Targeted CBT Approach for Motivational Negative Symptoms of Psychosis: Study Protocol for a Randomized-Controlled Feasibility Trial. Pilot Feasibility Stud..

[22] Grant P.M., Huh G.A., Perivoliotis D., Stolar N.M., Beck A.T. (2012). Randomized Trial to Evaluate the Efficacy of Cognitive Therapy for Low-Functioning Patients with Schizophrenia. Arch. Gen. Psychiatry.

[23] Moher D., Liberati A., Tetzlaff J., Altman D.G. (2009). Preferred Reporting Items for Systematic Reviews and Meta-Analyses: The PRISMA Statement. J. Clin. Epidemiol..

[24] Prinsen C.a.C., Mokkink L.B., Bouter L.M., Alonso J., Patrick D.L., de Vet H.C.W., Terwee C.B. (2018). COSMIN Guideline for Systematic Reviews of Patient-Reported Outcome Measures. Qual. Life Res..

[25] Mokkink L.B., de Vet H.C.W., Prinsen C.a.C., Patrick D.L., Alonso J., Bouter L.M., Terwee C.B. (2018). COSMIN Risk of Bias Checklist for Systematic Reviews of Patient-Reported Outcome Measures. Qual. Life Res..

[26] Terwee C.B., Mokkink L.B., Knol D.L., Ostelo R.W.J.G., Bouter L.M., de Vet H.C.W. (2012). Rating the Methodological Quality in Systematic Reviews of Studies on Measurement Properties: A Scoring System for the COSMIN Checklist. Qual. Life Res..

[27] Raugh I.M., Luther L., Bartolomeo L.A., Gupta T., Ristanovic I., Pelletier-Baldelli A., Mittal V.A., Walker E.F., Strauss G.P. (2023). Negative Symptom Inventory-Self-Report (NSI-SR): Initial Development and Validation. Schizophr. Res..

[28] Selten J.P., Sijben N.E., van den Bosch R.J., Omloo-Visser J., Warmerdam H. (1993). The Subjective Experience of Negative Symptoms: A Self-Rating Scale. Compr. Psychiatry.

[29] Park S.G., Llerena K., McCarthy J.M., Couture S.M., Bennett M.E., Blanchard J.J. (2012). Screening for Negative Symptoms: Preliminary Results from the Self-Report Version of the Clinical Assessment Interview for Negative Symptoms. Schizophr. Res..

[30] Engel M., Lincoln T.M. (2016). Motivation and Pleasure Scale-Self-Report (MAP-SR): Validation of the German Version of a Self-Report Measure for Screening Negative Symptoms in Schizophrenia. Compr. Psychiatry.

[31] Wang L.-L., Ma E.P.Y., Lui S.S.Y., Cheung E.F.C., Cheng K.S., Chan R.C.K. (2020). Validation and Extension of the Motivation and Pleasure Scale-Self Report (MAP-SR) across the Schizophrenia Spectrum in the Chinese Context. Asian J. Psychiatr..

[32] Kim J.-S., Jang S.-K., Park S.-C., Yi J.-S., Park J.-K., Lee J.S., Choi K.-H., Lee S.-H. (2016). Measuring Negative Symptoms in Patients with Schizophrenia: Reliability and Validity of the Korean Version of the Motivation and Pleasure Scale-Self-Report. Neuropsychiatr. Dis. Treat..

[33] Richter J., Hesse K., Eberle M.-C., Eckstein K.N., Zimmermann L., Schreiber L., Burmeister C.P., Wildgruber D., Klingberg S. (2019). Self-Assessment of Negative Symptoms—Critical Appraisal of the Motivation and Pleasure—Self-Report’s (MAP-SR) Validity and Reliability. Compr. Psychiatry.

[34] García-Portilla M.P., García-Álvarez L., de la Fuente-Tomás L., Dal Santo F., Velasco A., González-Blanco L., Zurrón-Madera P., Fonseca-Pedrero E., Bobes-Bascarán M.T., Sáiz P.A. (2021). Spanish Validation of the MAP-SR: Two Heads Better Than One for the Assessment of Negative Symptoms of Schizophrenia. Psicothema.

[35] Cernvall M., Bengtsson J., Bodén R. (2024). The Swedish Version of the Motivation and Pleasure Scale Self-Report (MAP-SR): Psychometric Properties in Patients with Schizophrenia or Depression. Nord. J. Psychiatry.

[36] Hervochon C., Bourgeois V., Rotharmel M., Duboc J.-B., Le Goff B., Quesada P., Campion D., Dollfus S., Guillin O. (2018). Validation of the French version of the self-evaluation of negative symptoms (SNS). Encephale.

[37] Wójciak P., Górna K., Domowicz K., Jaracz K., Szpalik R., Michalak M., Rybakowski J. (2019). Polish version of the Self-evaluation of Negative Symptoms (SNS). Psychiatr Pol..

[38] Hajj A., Hallit S., Chamoun K., Sacre H., Obeid S., Haddad C., Dollfus S., Rabbaa Khabbaz L. (2020). Validation of the Arabic Version of the “Self-Evaluation of Negative Symptoms” Scale (SNS). BMC Psychiatry.

[39] Mallet J., Guessoum S.B., Tebeka S., Le Strat Y., Dubertret C. (2020). Self-Evaluation of Negative Symptoms in Adolescent and Young Adult First Psychiatric Episodes. Prog. Neuro-Psychopharmacol. Biol. Psychiatry.

[40] García-Álvarez L., Martínez-Cao C., Bobes-Bascarán T., Portilla A., Courtet P., de la Fuente-Tomás L., Velasco Á., González-Blanco L., Zurrón-Madera P., Fonseca-Pedrero E. (2022). Validation of a European Spanish-Version of the Self-Evaluation of Negative Symptoms (SNS) in Patients with Schizophrenia. Rev. Psiquiatr. Salud. Ment..

[41] Tam M.H.W., Ling-Ling W., Cheng K., Wong J.O.Y., Cheung E.F.C., Lui S.S.Y., Chan R.C.K. (2021). Latent Structure of Self-Report Negative Symptoms in Patients with Schizophrenia: A Preliminary Study. Asian J. Psychiatry.

[42] Montvidas J., Adomaitienė V., Leskauskas D., Dollfus S. (2021). Validation of the Lithuanian Version of the Self-Evaluation of Negative Symptoms Scale (SNS). Nord. J. Psychiatry.

[43] Mazhari S., Karamooz A., Shahrbabaki M.E., Jahanbakhsh F., Dollfus S. (2021). Validity and Reliability of a Persian Version of the Self- Evaluation of Negative Symptoms (SNS). BMC Psychiatry.

[44] García-Álvarez L., Martínez-Cao C., Bobes-Bascarán T., Portilla A., Courtet P., de la Fuente-Tomás L., Velasco Á., González-Blanco L., Zurrón-Madera P., Fonseca-Pedrero E. (2022). Validation of a European Spanish-Version of the Self-Evaluation of Negative Symptoms (SNS) in Patients with Schizophrenia. Rev. Psiquiatr. Salud. Ment. (Engl. Ed.).

[45] Polat I., Ince Guliyev E., Elmas S., Karakaş S., Aydemir Ö., Üçok A. (2022). Validation of the Turkish Version of the Self-Evaluation of Negative Symptoms Scale (SNS). Int. J. Psychiatry Clin. Pract..

[46] Dollfus S., Mucci A., Giordano G.M., Bitter I., Austin S.F., Delouche C., Erfurth A., Fleischhacker W.W., Movina L., Glenthøj B. (2022). European Validation of the Self-Evaluation of Negative Symptoms (SNS): A Large Multinational and Multicenter Study. Front. Psychiatry.

[47] Samochowiec J., Jabłoński M., Plichta P., Piotrowski P., Stańczykiewicz B., Bielawski T., Misiak B. (2023). The Self-Evaluation of Negative Symptoms in Differentiating Deficit Schizophrenia: The Comparison of Sensitivity and Specificity with Other Tools. Psychopathology.

[48] Chen G., Chen J., Tian H., Lin C., Zhu J., Ping J., Chen L., Zhuo C., Jiang D. (2023). Validity and Reliability of a Chinese Version of the Self-Evaluation of Negative Symptoms. Brain Behav..

[49] Métivier L., Mauduy M., Beaunieux H., Dollfus S. (2024). Revealing the Unseen: Detecting Negative Symptoms in Students. J. Clin. Med..

[50] Overall J.E., Gorham D.R. (1962). The Brief Psychiatric Rating Scale. Psychol. Rep..

[51] Kay S.R., Fiszbein A., Opler L.A. (1987). The Positive and Negative Syndrome Scale (PANSS) for Schizophrenia. Schizophr. Bull..

[52] Andreasen N.C. (1989). The Scale for the Assessment of Negative Symptoms (SANS): Conceptual and Theoretical Foundations. Br. J. Psychiatry Suppl..

[53] Gard D.E., Gard M.G., Kring A.M., John O.P. (2006). Anticipatory and Consummatory Components of the Experience of Pleasure: A Scale Development Study. J. Res. Personal..

[54] Haro J.M., Kamath S.A., Ochoa S., Novick D., Rele K., Fargas A., Rodríguez M.J., Rele R., Orta J., Kharbeng A. (2003). The Clinical Global Impression-Schizophrenia Scale: A Simple Instrument to Measure the Diversity of Symptoms Present in Schizophrenia. Acta Psychiatr. Scand. Suppl..

[55] Axelrod B.N., Goldman R.S., Alphs L.D. (1993). Validation of the 16-Item Negative Symptom Assessment. J. Psychiatr. Res..

[56] Rodríguez-Testal J.F., Perona-Garcelán S., Dollfus S., Valdés-Díaz M., García-Martínez J., Ruíz-Veguilla M., Senín-Calderón C. (2019). Spanish Validation of the Self-Evaluation of Negative Symptoms Scale SNS in an Adolescent Population. BMC Psychiatry.

[57] Raffard S., Norton J., Van der Linden M., Lançon C., Benoit M., Capdevielle D. (2022). Psychometric Properties of the BIRT Motivation Questionnaire (BMQ), a Self-Measure of Avolition in Individuals with Schizophrenia. J. Psychiatr. Res..

[58] Tsui H.K.H., Wong T.Y., Sum M.Y., Chu S.T., Hui C.L.M., Chang W.C., Lee E.H.M., Suen Y., Chen E.Y.H., Chan S.K.W. (2024). Comparison of Negative Symptom Network Structures Between Patients with Early and Chronic Schizophrenia: A Network and Exploratory Graph Analysis. Schizophr. Bull..

[59] Strauss G.P., Ahmed A.O., Young J.W., Kirkpatrick B. (2018). Reconsidering the Latent Structure of Negative Symptoms in Schizophrenia: A Review of Evidence Supporting the 5 Consensus Domains. Schizophr. Bull..

[60] Li Y., Ang M.S., Yee J.Y., See Y.M., Lee J. (2024). Predictors of Functioning in Treatment-Resistant Schizophrenia: The Role of Negative Symptoms and Neurocognition. Front. Psychiatry.

[61] Mortimer A.M., McKenna P.J., Lund C.E., Mannuzza S. (1989). Rating of Negative Symptoms Using the High Royds Evaluation of Negativity (HEN) Scale. Br. J. Psychiatry Suppl..

[62] Torous J., Bucci S., Bell I.H., Kessing L.V., Faurholt-Jepsen M., Whelan P., Carvalho A.F., Keshavan M., Linardon J., Firth J. (2021). The Growing Field of Digital Psychiatry: Current Evidence and the Future of Apps, Social Media, Chatbots, and Virtual Reality. World Psychiatry.

[63] Wang L., Miller L.C. (2020). Just-in-the-Moment Adaptive Interventions (JITAI): A Meta-Analytical Review. Health Commun..

[64] Wild D., Grove A., Martin M., Eremenco S., McElroy S., Verjee-Lorenz A., Erikson P. (2005). Principles of Good Practice for the Translation and Cultural Adaptation Process for Patient-Reported Outcomes (PRO) Measures: Report of the ISPOR Task Force for Translation and Cultural Adaptation. Value Health.

[65] Page M.J., McKenzie J.E., Bossuyt P.M., Boutron I., Hoffmann T.C., Mulrow C.D., Shamseer L., Tetzlaff J.M., Akl E.A., Brennan S.E. (2021). The PRISMA 2020 statement: An updated guideline for reporting systematic reviews. BMJ.

